# Waste Minimization Protocols for the Process of Synthesizing Zeolites from South African Coal Fly Ash

**DOI:** 10.3390/ma6051688

**Published:** 2013-04-29

**Authors:** Pieter W. Du Plessis, Tunde V. Ojumu, Leslie F. Petrik

**Affiliations:** 1 Department of Chemical Engineering, Cape Peninsula University of Technology, Keizersgracht and Tennant street, Cape Town, 8000, South Africa; E-Mail: wynanddp@gmail.com; 2 Environmental and Nano Science Research Group, Department of Chemistry, University of the Western Cape, Private Bag X17, Bellville 7535, South Africa; E-Mail: lpetrik@uwc.ac.za

**Keywords:** zeolite Na-P1, zeolite analcime, fly ash, waste minimization, X-ray diffraction, atomic emission spectrometry, scanning electron microscopy

## Abstract

Production of a high value zeolite from fly ash has been shown to be an avenue for the utilization of South African fly ash which presently constitutes a huge disposal problem. The synthesis of zeolites Na-P1 and analcime on a micro-scale has been successful and preliminary investigation shows that scale-up synthesis is promising. However, the post-synthesis supernatant waste generated contains high levels of NaOH that may constitute a secondary disposal problem. A waste minimization protocol was developed to reduce the volume of waste generated with a view to enhancing the feasibility of the scale synthesis. Series of experiments were conducted in 100 mL jacketed batch reactors. Fly ash was reacted with 5 Mol NaOH on a 1:1 mass basis during the aging step, followed by hydrothermal treatment in which ultrapure water was added to the slurry. This study shows that by re-introducing the supernatant waste into the experiments in such a way that it supplies the required reagent (NaOH) for the zeolite synthesis, zeolite Na-P1 and analcime can be synthesized. It also shows that the synthesis process can be altered to allow up to 100% re-use of the supernatant waste to yield high value zeolitic products. This study effectively constructed two protocols for the minimization of waste generated during the synthesis of zeolites from South African coal fly ash. This result could be used to establish a basis for legal and environmental aspects involved in the commission of a full-scale plant synthesizing zeolites NaP1 and analcime.

## 1. Introduction

South Africa is largely dependent on coal as an energy source. In 2010, a projected total of 122.7 Mt of coal was consumed in Eskom coal fired power stations during electricity generation [1]. South Africa utilizes low grade coal, with high ash content, in the generation of electricity since high grade coal is exported. In 2010 Eskom generated 36 Mt of fly ash of which 34.16 Mt was disposed in ash dams and dumps [1].

Fly ash is comprised of fine, spherical glassy particles. The main constituents in fly ash are SiO_2_, Al_2_O_3_, Fe_2_O_3_ and CaO [2,3,4,5]. The majority of fly ash content are amorphous phases with the crystalline phases being quartz, mullite, hematite and magnetite [3]. To date, fly ash has found only low end uses such as an additive in cement [4]. It has also been used to neutralize and reduce sulphate content of acid and circumneutral mine waters [6,7]. However, due to the high Al and Si content of fly ash, it has been deemed a viable raw material for the synthesis of high value zeolites [8]. Numerous studies have investigated different types of methods to synthesize high value zeolites from coal fly ash [9,10,11,12,13]. However, the majority of these studies were performed on micro scale. 

It has been shown that a pure phase zeolite Na-P1 can be synthesized from South African coal fly ash using a two-step method, namely aging followed by hydrothermal treatment [11,12]. The authors reported the optimum conditions for the Aging and Hydrothermal steps to be 47 °C for 48 h and 140 °C for 48 h, respectively. The first activity in the scale up of a zeolite synthesis system is to optimize the process at micro scale. Since the composition of fly ash will differ from country to country and different power stations, it is also necessary to optimize the process for each fly ash [14]. Our recent study has shown the possibility to scale-up zeolite Na-P1 synthesis [15]. The authors synthesized a pure phase zeolite Na-P1 using the optimum condition reported previously [12], but at an ageing agitation rate of 200 rpm [15], using a four-blade impeller compared to 800 rpm reported by Musyoka et al using a magnetic stirrer [12].

However, for the successful scale up of the synthesis of high value zeolites from South African coal fly ash to be made possible, a clear understanding is required of the engineering design aspects of the synthesis system. One such critical aspect is the environmental concerns resulting from the waste generated through the process. It is important to characterize and quantify the waste generated from the process with a view to minimizing the waste by critical analysis of the processes involved in the synthesis of the target zeolitic product.

One of the waste products generated during the synthesis of zeolites Na-P1 from coal fly ash is the post synthesis supernatant which is separated from the zeolite product after hydrothermal treatment [11,12,15]. This supernatant consists of mainly sodium hydroxide and Si with trace amounts of heavy metals [16]. Since sodium hydroxide is a raw material utilized in the synthesis process, the opportunity exists to recycle the supernatant back into the synthesis process and effectively reduce the volumes of waste generated. It has been shown that zeolite Na-P1 can be synthesized from Chinese fly ash by reusing the waste supernatant generated [16], however the authors used a single step alkaline activation method with addition of sodium halide to promote the crystallization of the zeolitic product. The chemical composition of South African fly is vastly different; the Si/Al ratios are much less than that of the Chinese fly ash, thus making the single step method unsuitable for this fly ash. The differences in Si/Al ratios in different ashes results in a waste product rich in either Si or Al and thus changes the effects that recycling the waste solution has on the formed zeolitic product.

The aim of this study was to use the knowledge gleaned from previous studies to investigate waste minimization options for the synthesis of high value zeolites from South African coal fly ash. This study would explore the opportunity to recycle the post synthesis supernatant waste back into the synthesis system with a view to minimizing waste generation in a scale-up process which is currently being investigated.

## 2. Results and Discussion

### 2.1. Fly Ash Characterization

**Figure 1:**
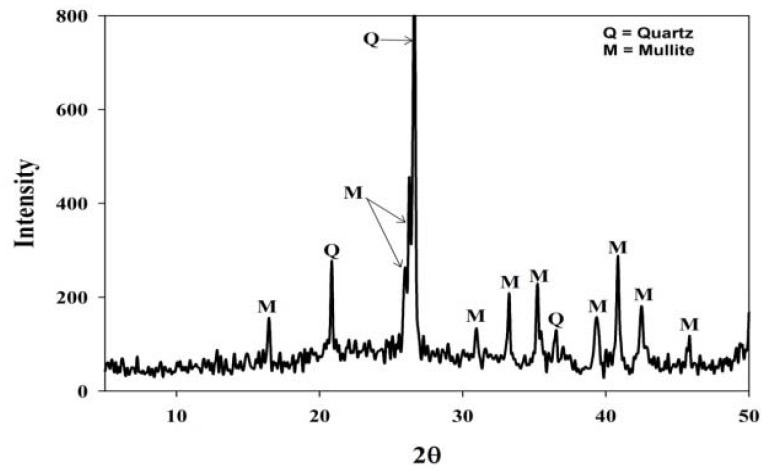
Powder X-ray diffraction patterns of coal fly ash from the Arnot power station.

From Figure 1 the XRD pattern indicates that the mineral phases in the fly ash consist of mainly quartz and mullite. In Figure 1 the broad hump, between 2θ = 20 and 2θ = 30, illustrates the amorphous phases in the ash which contributes more than 50% of the total mass. The mineralology of the ash greatly affects the zeolite synthesis mechanism since the different components in the ash dissolved with varying degrees of ease [17]. 

In the early stages of the study, analysis of the raw material revealed that the fly ash is classified as type F fly ash, in which the SiO_2_ + Al_2_O_3_ + Fe_2_O_3_ mass exceeds 70% of the total fly ash mass. Table 1 illustrates the major oxides and trace elements in Arnot fly ash as presented by X-Ray Fluorescence spectroscopy analysis (XRF). The SiO_2_/Al_2_O_3_ ratio was found to be 1.76 which is of great importance for the type of zeolite that will form using ash as a feedstock [10,18]. The ratio of Si to Al also determines what crystallization mechanism takes place during the formation of the zeolite crystals [19].Trace elements in the raw fly ash were monitored throughout the study to determine whether they report to the liquid waste or form part of the zeolite product as contaminants. Disposal of waste with high levels of these contaminants can have complex environmental consequences. 

**Table 1:** X-ray fluorescence results of Arnot coal fly ash composition, illustrating the quantities (wt%) of the major oxides and trace elements (ppm).

Major oxides (mean wt%)		Trace elemental concentrations (ppm)	
SiO_2_	55.44	Ba	486
Al2O_3_	31.51	Ce	254
Fe2O_3_	4.94	Co	30
MnO	0.03	Cu	110
MgO	1.18	Nb	37
CaO	3.76	Ni	125
Na_2_O	0.04	Pb	90
K2O	0.47	Rb	56
TiO_2_	1.11	Sr	989
P_2_O_5_	0.30	V	79
SO_3_	0.06	Y	94
Loss On Ignition	1.22	Zn	135
SiO_2_/Al_2_O_3_	1.76	-	-

### 2.2. Waste Recycle without a Prior pH Adjustment

**Figure 2:**
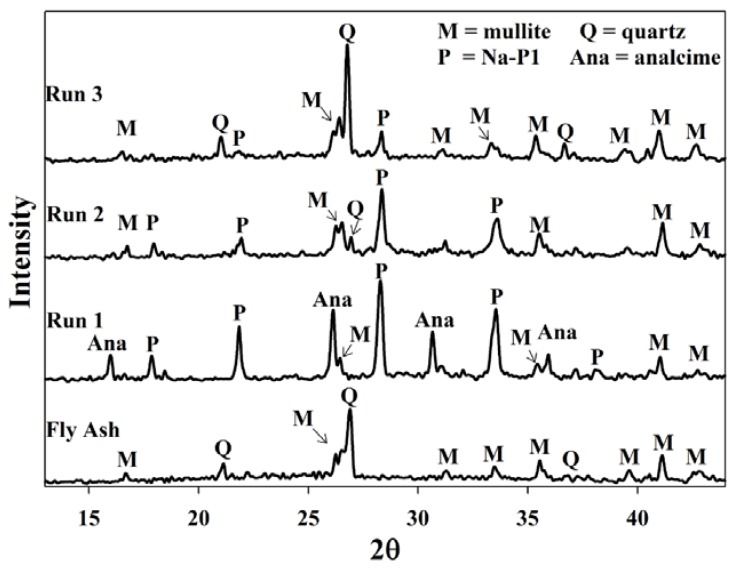
Powder X-ray diffraction patterns of products obtained after recycling waste supernatant back into the system as a NaOH source without a prior pH adjustment of the waste. **Run 1** Baseline run using fresh NaOH solution. **Run 2** synthesis using waste supernatant from Run 1 as NaOH source. **Run 3** synthesis using supernatant waste from Run 2 as NaOH source.

After the initial baseline experiment (Run 1), the supernatant waste was reused without adjusting its pH. XRD results (Figure 2) indicated that during Run 1 all of the quartz from the ash feedstock was dissolved. With small amounts of mullite the main crystal phases were found to be zeolite Na-P1 and analcime. After the first re-utilization of the supernatant (Run 2); it was observed that no analcime was formed, Na-P1 was reduced and less quartz and mullite dissolved. Also, after the second time of reusing the supernatant, only trace amounts of Na-P1 was found and almost none of the quartz and mullite were dissolved. The quality of zeolites formed and the dissolution of crystalline phases decreased after each successive run reusing the supernatant. The crystalline quantity of zeolite Na-P1 decreased from 55% in run 1, to a mere 17% in run 3. The reason being that after each successive run the pH of the supernatant decreased, resulting in poor mineralization of fly ash during reuse.

**Figure 3:**
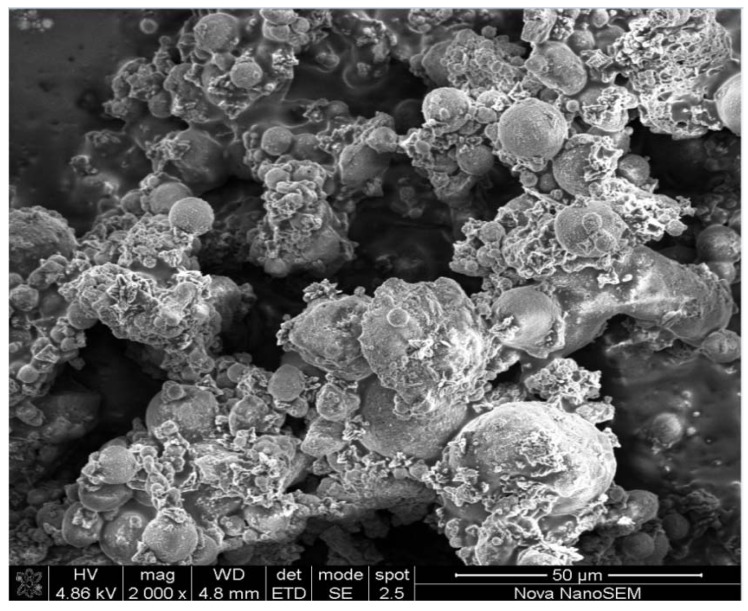
Micrographs taken of the products obtained after Run 3, at a magnification of 2000 times.

Figure 3 illustrates a micrograph taken of the resulting product after recycling the waste supernatant for a second consecutive time (Run 3). Results clearly indicate the spherically shaped, poorly dissolved fly ash particles as well as small traces of zeolite Na-P1. 

**Figure 4:**
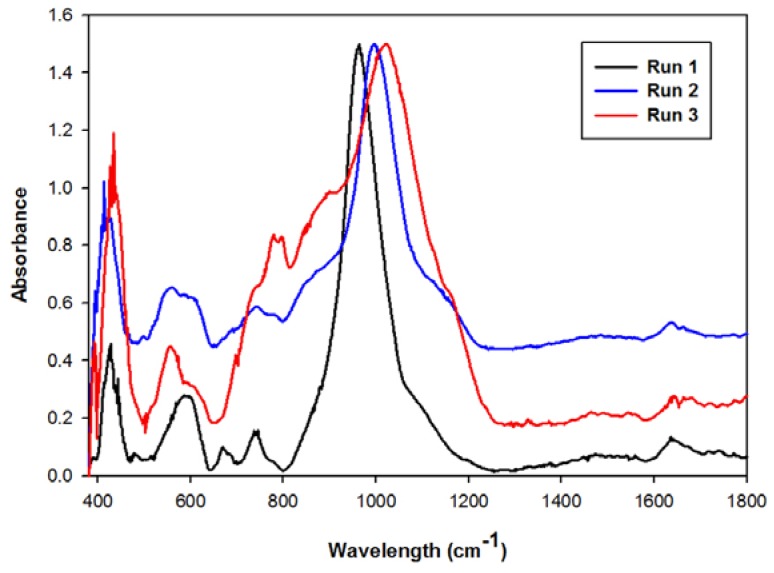
Infrared spectra of products obtained after recycling the supernatant waste as a source of NaOH.

From Figure 4 it is clear that the main absorption band in the 1000 cm^−1^ wavelength region shifted to the right from run 1 to run 3. This band represents the asymmetric stretching mode of the T-O (T = Si, Al) bonds that are found in the fly ash and zeolites [20,21]. This shift in the main band, from left to right, indicates a higher Si-O concentration in the crystal product [17]. This illustrates how less of the crystalline products in the original fly ash were dissolved and less zeolite products formed. Also, the bands appearing at around 700 and 800 cm^−1^ have been reported to be associated with the T-O (T = Al, Si) symmetric stretching vibrations that correspond to quartz present in the original fly ash starting material [21,22]. From these results it was clear that the pH of the waste supernatant would have to be adjusted prior to being recycled back into the synthesis system.

### 2.3. Waste Recycles with Prior pH Adjustment

**Figure 5:**
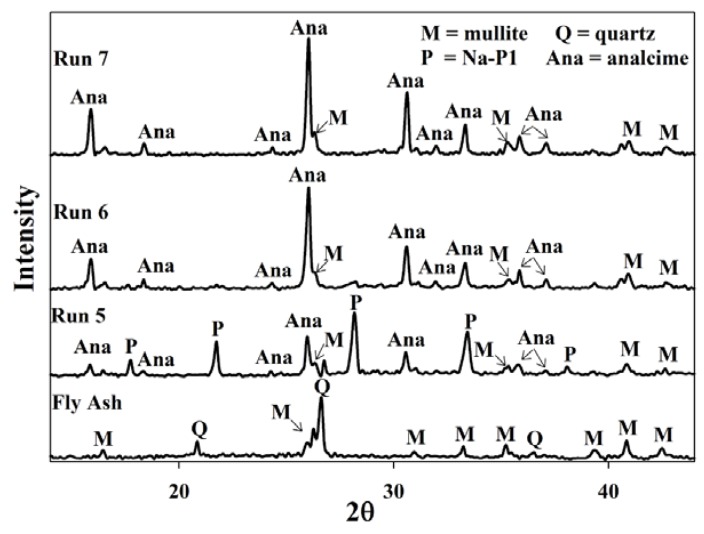
Powder X-ray diffraction patterns of products obtained after synthesis with supernatant waste as NaOH source after adjusting the pH of the waste. **Run 5** Baseline run using fresh NaOH solution. Run 6 synthesis using waste supernatant from Run 5 as NaOH source. Run 7 synthesis using supernatant waste from Run 6 as NaOH source.

In the second set of experiments once again the baseline experiment (Run 5 and Run 1) indicated the formation of both zeolites Na-P1 and analcime (Figure 5). However, by adjusting the pH of the supernatant before reusing it (Run 6), all the quartz and most of the mullite were dissolved. Also, after reusing the supernatant from run 6–7 zeolite analcime became more prominent and became the only crystalline phase present. The crystalline quantity of analcime increased from 36% in run 5 to 73% after run 7. One of the distinct differences between analcime and Na-P1 is the Si/Al ratio. While Na-P1 is a high Si/Al zeolite, analcime is a low Si/Al zeolite. The Al and Si content of the aging slurry are dependent on the dissolution of the amorphous phases and crystalline phases in fly ash. The dissolution process is complex due the fact that the different mineral phases in fly ash dissolve with different degrees of ease [17]. 

**Figure 6:**
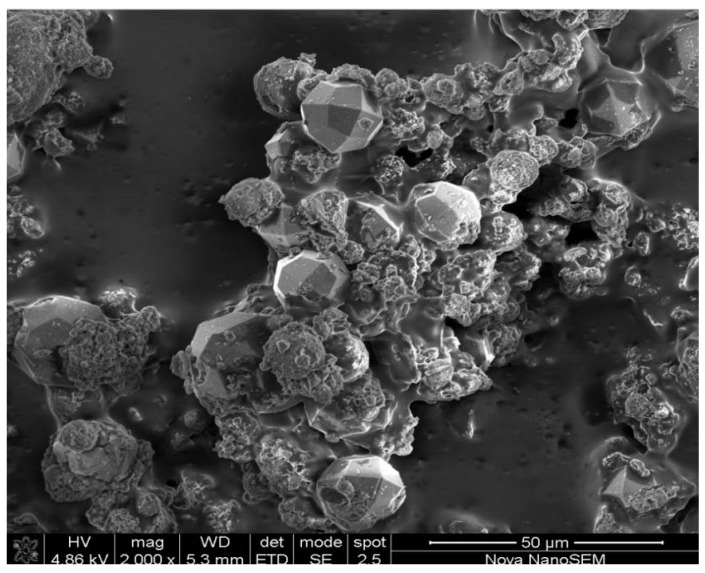
Micrographs taken of the products obtained after Run 7, at a magnification of 2000 times.

Figure 6 illustrates a micrograph, magnified 2000 times, of the product obtained after the second consecutive recycle of the waste supernatant. The product contains mostly analcime but traces of amorphous material could also be observed. This agrees with the crystallinity results of 73%, and can possibly be increased by optimizing the hydrothermal treatment time.

**Figure 7:**
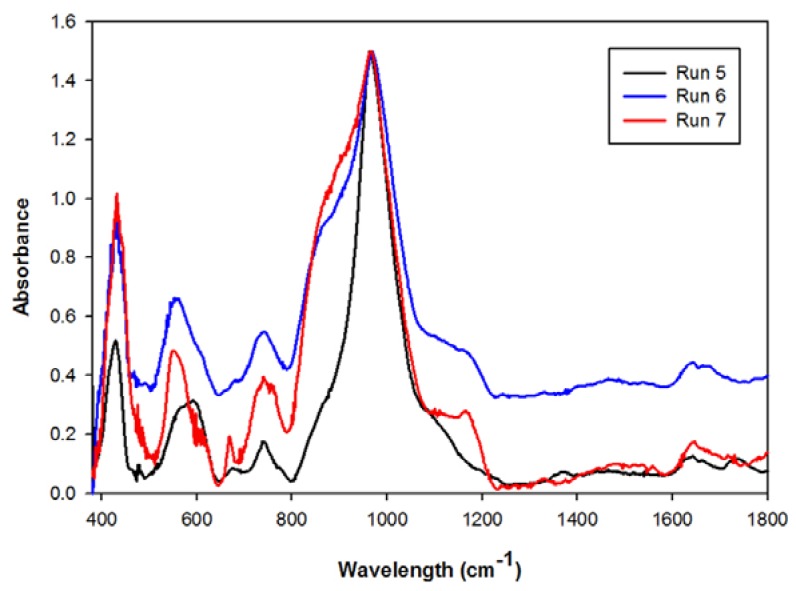
Infrared spectra of products obtained after recycling the supernatant waste as a source of NaOH.

From Figure 7 it can be seen that the main absorption band in the 1000 cm^−1^ wavelength region did not shift its position considerably. The minor, almost unnoticeable shift in its position was due to the fact that the Al-O and Si-O concentrations in the two zeolite structures, analcime and Na-P1, differs [20,23]. The increase in the band intensities in the 650 and 750cm^−1^ regions, as well as the hump illustrated in the 900 cm^−1^ region, illustrates analcime becoming the more dominant phase in the product [24].

In order to establish a basis for environmental and legal requirements for the disposal of waste solutions, the concentrations of the major species found in the waste solutions were determined. Table 2 illustrates the concentrations of these species as determined by inductively coupled plasma atomic emission spectrometry (ICP-AES). From the results it was clear that all the elemental concentrations in the waste solution increased as the waste was reused, indicating that there is an accumulation of these species. However, aluminum did not accumulate and was found to be the limiting element for zeolite growth. It is clear that the accumulation of Si as the waste was reused favored the formation of analcime [25]. This indicated that in order to further increase the crystallinity of the final analcime product, both crystallization time and the Si/Al ratio of the reacting slurry needs to be optimized. With a pore size of about 0.25 nm, zeolite analcime can be use in the absorption of NH_3_ molecules [20]. Removal and recovery of these elements from solution would not be a viable option since its low concentration would make such an option un-economical. The trace elements from the fly ash do not affect the zeolite product during the synthesis process. This was found to be a result of their low mobility due to the CaO content in fly ash [26]. Thus the majority of the elements do not leach out into solution during the dissolution of the Si and Al species from the ash. 

**Table 2:** Atomic emission spectrometry results indicating elemental concentrations of major species found in the waste supernatant after each successive run.

Mean elemental concentration (ppm)			
Element	Run 5	Run 6	Run 7
Al	35.5	34.0	31.3
Fe	5.3	6.9	7.4
K	215.1	434.6	497.2
Na	20971.3	25348.0	26101.4
P	80.6	163.5	196.8
Si	7198.7	15124.7	16624.1
Ti	4.9	11.3	12.8
V	5.1	9.8	11.3

### 2.4. Recycling 100% Waste Supernatant

**Figure 8:**
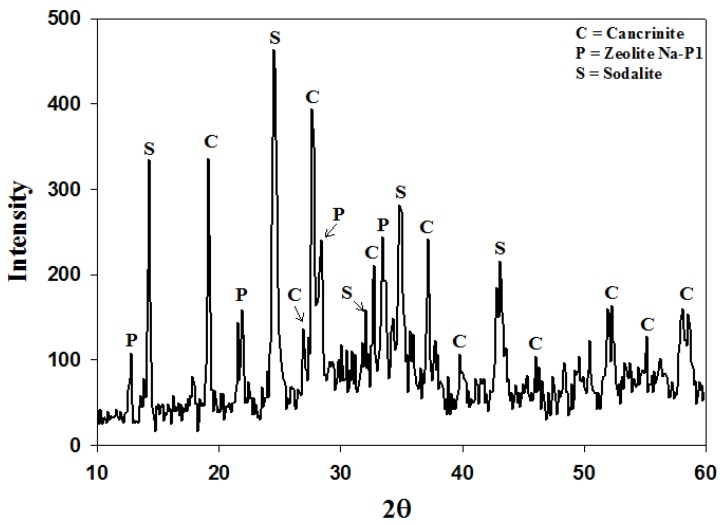
XRD patterns of the resulting product after removing the water addition step during the synthesis of zeolites from coal fly ash.

**Figure 9:**
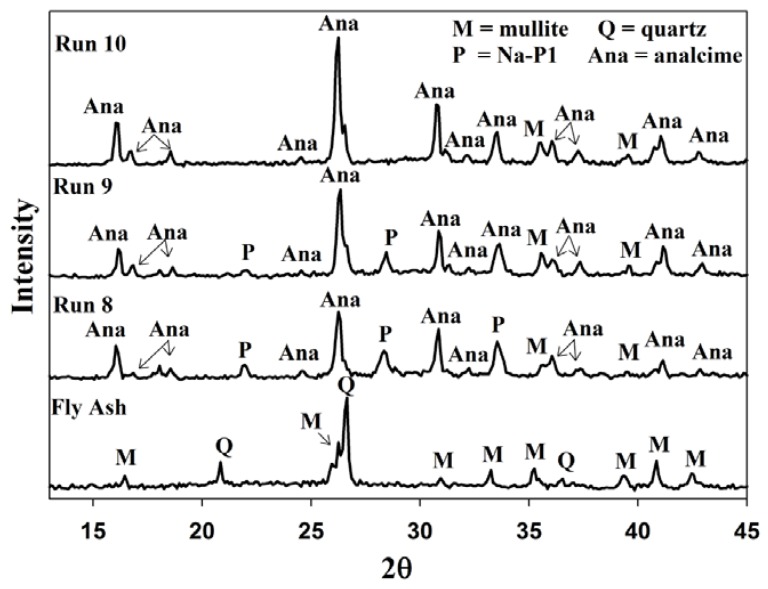
Powder X-ray diffraction patterns of products obtained after synthesis with supernatant waste as NaOH source using the amended synthesis approach. **Run 8** Baseline run using fresh NaOH solution. **Run 9** synthesis using waste supernatant from Run 8 as NaOH source. **Run 10** synthesis using supernatant waste from Run 9 as NaOH source.

The waste minimization protocol discussed in Section 2.3 could only recycle 40% of the waste supernatant. The reason for this is that the process starts off with a 5 M NaOH solution and after the aging step, ultrapure water, equal to 150% by volume of the starting 5 M NaOH solution, is added to the reacting slurry. Thus the volume of waste generated far exceeds the volume of NaOH in the feedstock. This water addition step could not be omitted completely. When using fly ash as a feedstock in zeolite synthesis, the liquid volumes need to be kept at a much higher level than in commercial synthesis. The reason for this is that an increase in water content increases the dissolution of Al and Si species from fly ash, and favors the existence of metastable species [27,28]. When the water addition step is omitted completely a poorly crystallized product was obtained with various contaminating phases. Figure 8 illustrates the XRD patterns of the resulting product when this attempt was made. Three different crystalline phases were found in the product namely zeolite Na-P1, sodalite and cancrinite. The two broad humps in the angle ranges of 20 < 2θ < 40 and 2θ > 50 indicates undissolved amorphous phases. The crystallinity of zeolite Na-P1, cancrinite and sodalite was found to be 26%, 12% and 7% respectively. The majority of the sample was found to exist in an amorphous state. This poor formation of metastable phases highlighted the importance of using higher water content when using ash as a feedstock. In order to remedy this bottleneck in the system an attempt was made to introduce the addition of ultrapure water at a much earlier stage before the aging stage commences. This step kept the process liquid volume constant throughout the different synthesis stages. The consequence of this was that the starting NaOH solution would have a molarity of 2 M, but 100% of the waste could be introduced into the system without a pH adjustment. In the process of aging fly ash in an alkaline solution a decrease in pH results in a decrease in the rate of quartz dissolution [29] and also in the rate of hydrolysis of the glassy phases [30]. However, Figure 9 depicts that the quartz and mullite did indeed dissolve with only traces of mullite visible after ultrapure water was introduced before the aging step. From the first set of Runs (Run 8) it can be seen that the two major crystalline phases formed were zeolites Na-P1 and analcime. As the supernatant waste was reused from run 8 to 10, the amount of analcime increased from 80% to a near pure phase. This trend is similar to that seen in Figure 5. 

**Figure 10:**
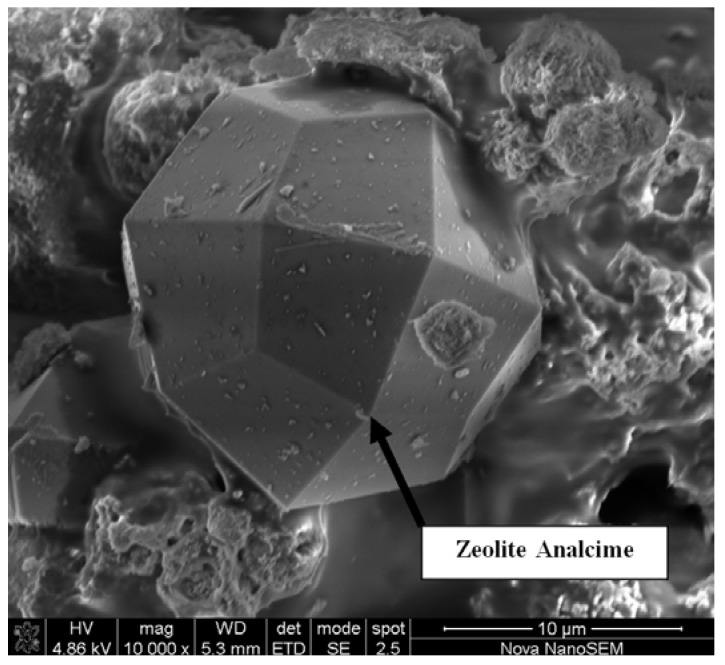
Micrographs taken of zeolite analcime obtained after Run 10, at a magnification of 10,000 times.

A micrograph of the zeolite analcime crystals obtained, magnified 10,000 times, illustrate the well defined faces and crystal structure of the analcime product (Figure 10). The analcime crystal can be described as a trapezohedron shape, with a total of 24 faces.

By means of ICP-AES analysis the concentrations of the elements in the final waste solution were obtained. The concentrations of Al, Fe, K, Na, P, Si, Ti and V were found to be 25.9, 11.6, 550.4, 25,803.5, 237.9, 19,783.2, 14.1 and 13.2 ppm respectively. The concentrations of the elements were found to be in the same range as the data for the second recycle (Run 7) in Table 2. This illustrates that the same rate of accumulation among the elements exists in the two different synthesis processes. However, a much higher Si concentration exists because this protocol recycles 100% of the Si in the waste back into the system.

This alteration in the basic synthesis process illustrated great success in significantly reducing the amount of waste produced by the process and consequently the cost of disposal. By reducing the waste produced from this process the feasibility of a scale up operation has been reinstated and can be applied to relieve the difficulties faced with the generation of coal fly ash in South Africa. 

**Figure 11:**
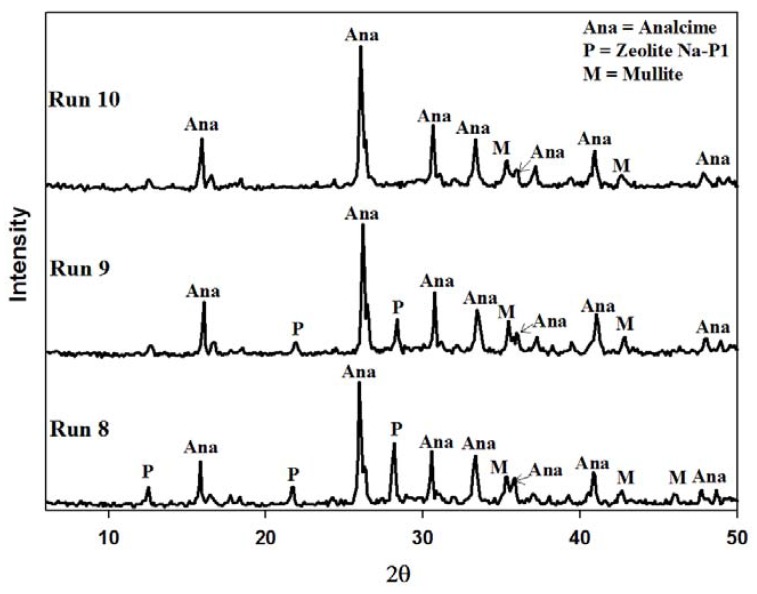
XRD patterns illustrating reproducibility of results depicted in figure 8 (Second results set).

**Figure 12:**
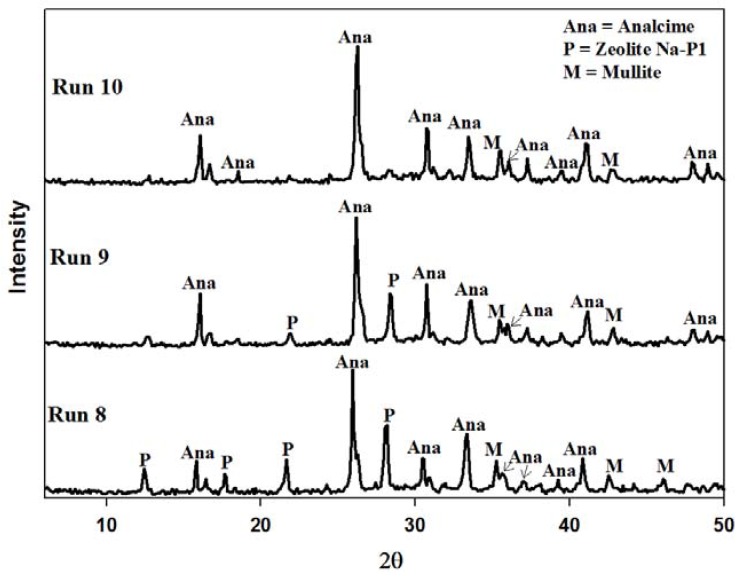
XRD patterns illustrating reproducibility of results depicted in Figure 8 (third results set).

Figure 11 and Figure 12 illustrate the reproducibility of this new synthesis approach. It was found to be highly reproducible with the crystallinity of analcime, in all cases, increasing from ±75% to near 100% after the second recycle of the supernatant waste. In all cases the average product yield was found to be 9.7 g from an ash feed of 10 g, while the space time yield was found to be 14.5 kg m^−3^ day^−1^.

## 3. Experimental Section

### 3.1. Materials and Characterization

The fly ash used in this study was supplied by a power station situated in Mpumalanga, South Africa. Analytical grade sodium hydroxide pellets were chosen for the synthesis of zeolites from fly ash. For the preparation of fresh NaOH solution and the washing of zeolite products, ultra pure water was used. The elemental composition of raw material was determined by performing X-ray fluorescence (XRF) spectroscopy, while elemental concentrations in liquid products were determined with inductively coupled plasma atomic emission spectrometry (ICP-AES). The mineralogy of all solid products and raw ash was revealed by means of X-ray powder diffraction using Cu-Kα radiation in a range of 4 < 2θ < 60. Changes in zeolite structure were observed by means of attenuated total reflectance Fourier transform infrared spectroscopy (ATR-FTIR). The morphology of zeolitic products was observed by means of scanning electron microscopy (SEM).

### 3.2. Synthesis of Zeolites from Coal Fly Ash

The zeolite synthesis procedure was adopted from Musyoka [12] which consists of two steps *i.e.*, aging followed by hydrothermal treatment. 

**Figure 13:**
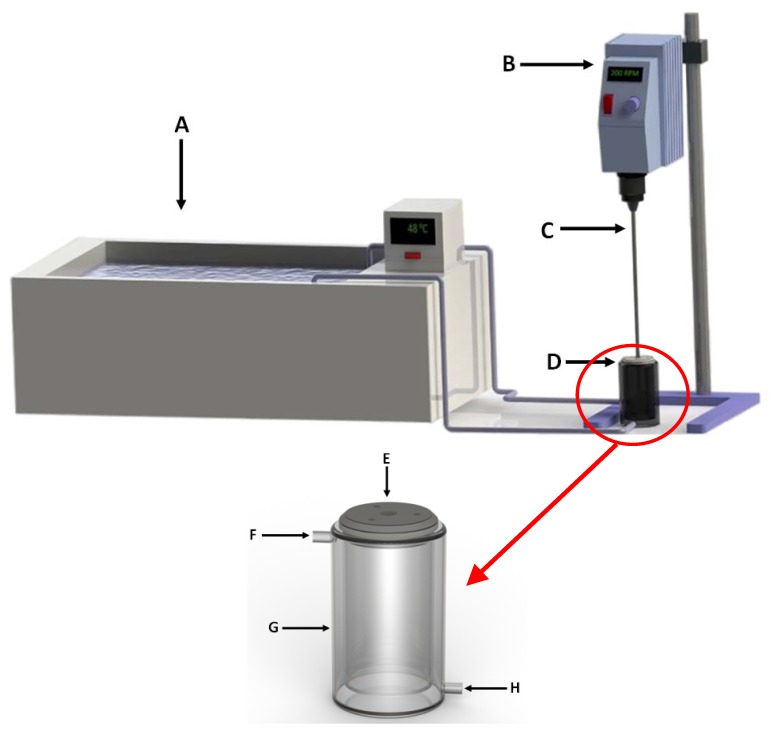
Experimental setup for aging step (**A**) Water bath; (**B**) Variable speed mixer; (**C**) 4-blade paddle impeller; (**D**) Double walled glass reactor; (**E**) Teflon reactor lid; (**F**) Heating water inlet; (**H**) Heating water outlet; (**G**) Double walled glass body.

The aging step was performed in a 100 mL double walled glass reactor connected to a water bath which served as heat source to maintain the aging medium at 47 °C (Figure 13). The aging medium was mixed utilizing a 4-blade paddle impeller at 200 rpm. After 50 mL NaOH solution of 5 M was prepared and preheated inside the 100 mL glass reactor, aging was initiated by adding 10 g of fly ash to the reactor. The aging step then proceeded for 48 hours. 

**Figure 14:**
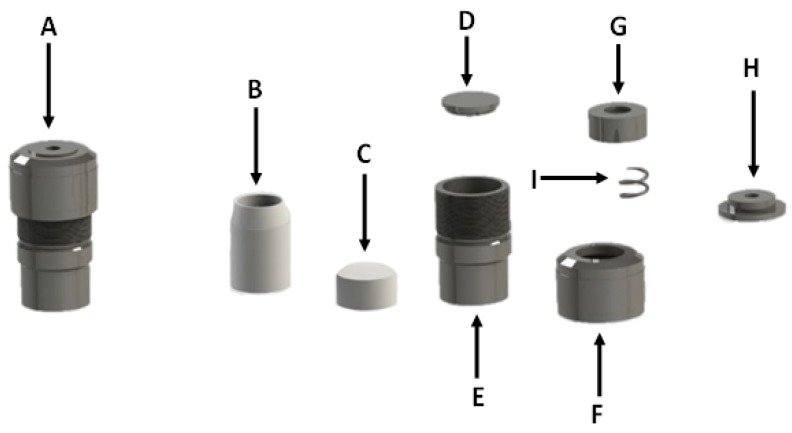
Parr bomb reactor assembly (**A**) Assembled reactor; (**B**) Teflon lining; (**C**) Teflon lid; (**D**) Bottom metal plate; (**E**) Metal casing; (**F**) Metal lid; (**G**) Metal weight keeping teflon lid in place; (**H**) Top metal plate; (**I**) Spring separating metal weight and top plate.

After the aging step, 75 mL of ultrapure water was added to the aged medium under agitation, after which the mixture was transferred into 23 mL Teflon lined autoclave reactors (Figure 14). The autoclave reactors were placed in a hot air oven at 140 °C in order for the hydrothermal treatment stage to commence. The hydrothermal stage proceeded for 48 hours. After hydrothermal treatment the products were separated from the post synthesis supernatant after which it was washed and dried.

### 3.3. Research Structure

Table 3 outlines the experimental approach followed during this investigation. After initial runs were completed using fresh NaOH (Run 1, 5 and 8), the post supernatant was recycled. First the supernatant was used without adjustments to establish a starting base for the research (Runs 2–3). After these set of experiments were completed the supernatant was titrated to determine the OH^−^ ion concentration. With this data in hand, the concentration of NaOH in the waste was adjusted to match that of the fresh 5 M solution, by addition of NaOH pellets, before being recycled (Runs 6–7). In each set of experiments the supernatant was recycled twice. With this protocol, a total of 40% liquid waste could be recycled. In order to recycle 100% of the waste the starting liquid volume should be similar to the waste volume. This was achieved in the following set of experiments (Runs 8–10) whereby the 75 mL post aging water was added at the start of the aging process, thus changing the NaOH concentration from 5 M to 2 M. In doing so, 100% could be recycled without the need to adjust the alkalinity.

**Table 3:** Experimental structure.

Run	Process description	Adjustments to alkali source
1	Synthesis of zeolites using 50 mL of a fresh 5 M NaOH batch as alkali source	-
2	Utilizing the supernatant waste generated from run 1 as alkali source	None
3	Utilizing the supernatant waste generated from run 2 as alkali source	None
4	Repetition of reference run (Run 1) to generate supernatant for titration purposes	-
5	Synthesis of zeolites using 50 mL of a fresh 5 M NaOH batch as alkali source	-
6	Utilizing the supernatant waste generated from run 5 as alkali source	pH adjusted
7	Utilizing the supernatant waste generated from run 6 as alkali source	pH adjusted
8	Synthesis of zeolites using 125 mL of a fresh 2 M NaOH batch as alkali source	-
9	Utilizing the supernatant waste generated from run 8 as alkali source	None
10	Utilizing the supernatant waste generated from run 9 as alkali source	None

## 4. Conclusions

Waste minimization options for the synthesis of zeolites from South African coal fly ash were investigated. The opportunity to recycle 40% of the waste supernatant back into the system illustrated that to successfully synthesize zeolites with the waste solution, its pH needs adjustment. By adjusting the pH of the supernatant prior to being reused, zeolites analcime and Na-P1 were successfully synthesized while dissolving unwanted crystalline minerals from fly ash. Zeolite analcime was found to be the dominant phase after reusing the supernatant waste due to a high Si/Al ratio in the waste. By altering the basic synthesis process slightly it was also possible to recycle 100% of the supernatant waste without an adjustment to its alkalinity. This study effectively developed two protocols in which waste supernatant can be reduced drastically and thereby improving the scale-up feasibility. It is recommended that future studies should investigate altering the Si/Al ratio of both the fly ash and supernatant to obtain the more desirable zeolite Na-P1 as dominant zeolite phase. 
